# Levels of empathy among undergraduate physiotherapy students: A cross-sectional study at two universities in Istanbul

**DOI:** 10.12669/pjms.321.8745

**Published:** 2016

**Authors:** Hulya Yucel, Gonul Acar

**Affiliations:** 1 Hulya Yucel, PT, PhD, Assistant Professor, Department of Ergotherapy, Faculty of Health Sciences, Bezmialem Vakif University, Istanbul, Turkey; 2 Gonul Acar, PT, PhD, Assistant Professor, Department of Physiotherapy and Rehabilitation, Faculty of Health Sciences, Marmara University, Istanbul, Turkey

**Keywords:** Empathy, Physiotherapy students, Curriculumn, Jefferson scale of physician empathy

## Abstract

**Objective:**

The aim of this study is to determine differences of levels of empathy among undergraduates in each year of their four-year programs of physiotherapy.

**Methods:**

During the 2014-2015 academic school year, 381 physiotherapy students were enlisted from two universities in Istanbul, one a foundation and the other a government university. The Turkish version of the Jefferson Scale of Physician Empathy was administered. Students were asked to indicate interest in particular physiotherapy specialties as well as their region of origin in Turkey. The Kruskal-Wallis analysis was used to determine differences among the four study years, and also to measure relationships between specialty interest, home-region, and empathy scores of the students. Empathy scores were also compared according to gender.

**Results:**

The difference of empathy scores between the students of the two universities was borderline significant (p=0.057). Empathy scores in both universities increased to a significant degree after school entrance and decreased in the final year. Levels of empathy did not change according to gender, specialty interest, or home-region (p=0.722, 0.524, and 0.309, respectively).

**Conclusions:**

This study points to the need for physiotherapy curricula that would enhance empathy and give students practice in exhibiting this valuable attribute. Additional studies are needed that would include larger study populations and track the same students year by year as to how and why their empathy levels change during their training.

## INTRODUCTION

Empathy is the ability to consider a situation from someone else’s point of view, to understand the feelings of others, and to communicate this understanding to them.^1^ Factors that may affect empathy include age, family background, culture, intelligence, education, personality, and, specialty interests.^2^,^3^

Empathy is an important attribute for health care providers in general because it is associated with improved clinical outcomes.^4^ Patients who feel understood are more likely to be able to clearly explain their complaints.^5^ In the literature, studies have compared levels of empathy of health professionals from various departments such as occupational therapy, nursing, medicine, and physiotherapy.^6^-^8^ In physiotherapy, particularly, interpersonal skills and concern for others are of key importance.

In Turkey, the current curriculum for physiotherapists does not specifically address empathy. The first year of the 4-year training program covers basic sciences, while the second and especially third years concentrate on clinical assessments and treatments. The final year is primarily an internship. Because empathy is so important, it should be specifically incorporated as a part of the training curricula, for example in psychosocial rehabilitation, a second-year course that deals with people who have various diseases and handicaps. More empathy-based education and more empathetic educators positively affect student’s levels of empathy. Students spend hours with a clinical educator, watching his behavior, and emulating what they observe how to approach patients.^9^,^10^ Because of the difference of instructors, levels of empathy may differ between students at different institutions, even those which share similar curricula and student demographics.^11^

Research on empathy among health care professionals has been done in many countries,^7^,^8^,^12^,^13^ but to the best of our knowledge, no study in the literature examines and compares each of the four years of students’ physiotherapy training. Ours is the first to do so. Our aim was to analyze self-reported levels of empathy of undergraduate physiotherapy students from two universities, and also determine any differences among their four different years of study.

## METHODS

This study assessed levels of empathy of physiotherapy students from two universities in Istanbul: Bezmialem Vakif University (BVU), a foundation university, and Marmara University (MU), a government university. Participants were in either the first, second, third, or fourth year of their respective programs during the 2014-2015 academic year. Out of the possible total of 431 students, 381 consented to take part (102 from BVU and 279 from MU). The study was approved by the Institutional Review Board from BVU (71306642/050-01-04/257, 17.09.2014) and performed in accordance with the Declaration of Helsinki once participants had given their oral informed consent.

After permission from their instructors, each student received a ten-minute written questionnaire to complete anonymously. They provided information related to age, gender, and study year, and were then asked to indicate the particular field of physiotherapy in which they would most prefer to specialize, i.e., gerontology, women’s health, pediatric rehabilitation, neurologic rehabilitation, orthopedic rehabilitation, sportsmen’s rehabilitation, cardiopulmonary rehabilitation, hand rehabilitation, or other (such as oncology).^3^,^12^ To account for cultural variation of empathy, students marked if they were foreign nationals, or from which of the seven major regions in Turkey they came from, i.e., The Mediterranean, The Aegean, The Marmara, The Black Sea, The East Anatolia, The Southeastern Anatolia, or Central Anatolia.

In the literature to measure empathy, the most commonly used quantitative method is the Jefferson Scale of Physician Empathy (JSPE), developed in response to the need for a content-specific and context-relevant instrument to measure empathy.^1^,^2^ The JSPE has been translated into 39 languages, and studies have confirmed its validity in Mexico, Korea, Iran, and the United Kingdom.^1^,^8^,^13^ According to factor loadings, R^2^, and goodness-of-fit statistics, a three-factor structure was confirmed for the student version of the JSPE. The internal consistencies of the components were adequate at the factor level for the “perspective taking”, “compassionate care”, and “standing in the patient’s shoes” components (Cronbach’s alphas were 0.83, 0.70 and 0.60, respectively).^2^ Although the JSPE was originally designed for students in medicine^2^, it was later modified for use among practicing physicians and other health professionals in general.^1^,^2^ Both versions are currently available. One is applicable to physicians and other practicing health professionals; the second applies to students in those fields. That is student version is applicable to students in medical and other health professsions.^2^ For instance; Fjortoft et al. showed spesific uses of the JSPE for pharmacy students in the assessment of educational outcomes of different pharmacy programs to enhance student empathy, in research on correlates of empathy in pharmacy education and practice, and in group comparisons within the pharmaceutical discipline as well as between pharmacy and other health disciplines.^14^ To examine the self-reported empathy levels of those in our study population, we administered the Turkish version of the JSPE for medical students adapted from Hojat et al.^1^ which had been reworded so as to apply specifically to physiotherapy students. For example, in the item “Physicians should try to stand in their patients’ shoes when providing care for them”, “physicians” was replaced with “physiotherapists”.

The JSPE contains 20 items, each to be answered on a seven-point Likert-type scale. Half of the items were positively worded (items 2, 4-5, 9-10, 13, 15-17, 20) and directly scored from 1 (strongly disagree) to 7 (strongly agree). The remainder (items 1, 3, 6-8, 11-12, 14, 18-19) were negatively worded and scored in reverse, from 1 (strongly agree) to 7 (strongly disagree). The total score was obtained by summing all items. The score range is 20–140, with higher mean scores representing higher levels of empathy.^1^,^2^

### Statistical analysis

Statistical analysis of the study was performed with SPSS v.23 (SPSS Inc., Chicago, IL, USA). Frequencies are shown as number (n) and percentage (%). In power analysis, when the JSPE scores of two groups were considered in the literature, the 153 participants (51 students from BVU and 102 from MU) resulted in a α = 0.05 significance level and 80% power. Since this is a survey study, a number more than 153 was taken in order to obtain more powerful findings. P<0.05 was accepted as significant.

Various statistical tests were used depending on the variables being analyzed. The Mann-Whitney U test was used for non-parametric and artificial quantitative variables. Median and minimum-maximum (min-max) values were recorded. Empathy scores according to gender were compared. The Kruskal-Wallis one-way analysis of variance was used for differences among the four study years. The Bonferroni test correction was made to determine where the difference arises from among the study years. P value was 0.008 (0.05/the number of comparisons). The Kruskal Wallis test was used to measure relationships between field of work, home-region, and empathy scores of the students.

## RESULTS

Of the study participants, 102 (26.8%) students were from BVU and 279 (73.2%) were from MU. Their mean age was 20.7±1.3 (17-24) years. Among them, 165 (43.3%) were first year students, 82 (21.5%) were second year, 64 (16.8%) were in their third year, and 70 (18.3%) were final year students.

Distributions of the students’ gender, field of work, and home-region are shown at Table-I. The majority were female (272, 71.4%). Both the median female and male empathy scores were 111.0. There was no statistically significant difference between them (p=0.722). In both universities, students primarily wanted to work in either pediatric rehabilitation or neurologic rehabilitation. No relationship arose between field of work and empathy scores (p=0.524). By home-region, slightly more than one-fourth of all students came from the Black Sea region of Turkey (106, 27.8%). No statistically significant relationship surfaced between home-region and empathy scores (p=0.309).

**Table-I:** Distributions of the students’ gender, field of work, and home-region

	BVU n (%)	MU n (%)	Total n (%)
*Gender*			
Male	22 (21.6)	86 (30.8)	108 (28.3)
Female	80 (78.4)	193 (69.1)	273 (71.7)
*Field of work*			
Gerontology	6 (5.9)	10 (3.6)	16 (4.2)
Women’s Health	19 (18.6)	26 (9.3)	45 (11.8)
Paediatric Rehabilitation	34 (33.3)	97 (34.8)	131 (34.4)
Neurologic Rehabilitation	17 (16.7)	51 (18.3)	68 (17.8)
Orthopaedic Rehabilitation	11 (10.8)	36 (12.9)	47 (12.3)
Sportsmen’s Rehabilitation	6 (5.9)	25 (9.0)	31 (8.1)
Cardiopulmonary Rehabilitation	1 (1.0)	7 (2.5)	8 (2.1)
Hand Rehabilitation	1 (1.0)	3 (1.1)	4 (1.0)
Others	4 (3.9)	18 (6.5)	22 (5.8)
Unspecified	3 (2.9)	6 (2.2)	9 (2.4)
*Home-region*			
East Anatolia	13 (12.7)	36 (12.9)	49 (12.9)
Southeastern Anatolia	13 (12.7)	31 (11.1)	44 (11.5)
Mediterranean	7 (6.9)	23 (8.2)	30 (7.9)
Agean	3 (2.9)	32 (11.5)	35 (9.2)
The Marmara	17 (16.7)	36 (12.9)	53 (13.9)
Black Sea	31 (30.4)	75 (26.9)	106 (27.8)
Central Anatolia	14 (13.7)	40 (14.3)	54 (14.2)
Foreign Nationality	1 (1.0)	4 (1.4)	5 (1.3)
Unspecified	3 (2.9)	2 (0.7)	5 (1.3)

Total	102 (100)	279 (100)	381 (100)

BVU: Bezmialem Vakif University; MU: Marmara University, n: number; %: percentage

The difference of empathy scores of all students related to study years was significant (p<0.001) (Table-II). This arose from the differences between 1st-2nd, 1st-3rd, 2nd-4th, and 3rd-4th study years.

**Table-II:** Empathy scores of all students related to study years

Study years (n)	Empathy scores median (min-max)	p*	p**
1 (165)	108.0 (59.0-135.0)	p<0.001	1-2:0.001
2 (82)	114.0 (72.0-137.0)		1-3:0.001
3 (64)	116.5 (90.0-140.0)		1-4:0.092
4 (70)	108.0 (51.0-131.0)		2-3:0.567
			2-4:<0.001
			3-4:<0.001

n: number; min: minimum; max: maximum;

* p: the difference among study years;

** p: the difference shows in which study year differences arise.

Differences between BVU and MU students, between their study years of both universities seperately, and among study years at each university are shown at Table-III. According to the total scores, MU students had lower empathy values than those at BVU. The difference of total empathy scores between two universities was borderline significant (p=0.057); however, at both institutions, statistically significant differences surfaced at the first and last study years (<0.001 and 0.002, respectively), with a significant increase after the first study year, but a marked decrease in the fourth. Levels of empathy differences among study years at each university separately were significant (p values were 0.001 at BVU and 0.013 at MU). The differences arose from the differences between the 1st-4th, 2nd-4th, and 3rd-4th study years at BVU and between the 1st-2nd and 1st-3rd study years at MU.

**Table-III:** Empathy scores related to two universities

Study years (n)	Empathy scores		p*

	BVU	MU	

	median (min-max) (n)	median (min-max) (n)	
1 (165)	117.0 (99.0-129.0) (36)	107.0 (59.0-135.0) (129)	<0.001
2 (82)	118.5 (89.0-135.0) (22)	114.0 (72.0-137.0) (60)	0.096
3 (64)	121.0 (90.0-137.0) (19)	115.0 (95.0-140.0) (45)	0.941
4 (70)	82.0 (51.0-131.0) (25)	110.0 (80.0-130.0) (45)	0.002
Total (381)	114.5 (51.0-137.0)(102)	110.0 (59.0-140.0) (279)	0.057
p**	0.001	0.013	
p***	1-2:0.608	1-2:<0.001
	1-3:0.750	1-3:<0.001
	1-4:<0.001	1-4:0.22
	2-3:0.666	2-3:0.329
	2-4:<0.001	2-4:0.088
	3-4:0.001	3-4:0.013

BVU: Bezmialem Vakif University; MU: Marmara University, n: number; min: minimum; max: maximum;

* p: the difference between study years in both universities;

** p: the difference between study years in a university;

*** p: the difference shows in which study years differences arise

## DISCUSSION

This study is the first to assess the levels of empathy among undergraduate physiotherapy students in Turkey. Our results showed that the empathy scores increased slightly after school entrance and decreased significantly in the final year.

In the literature, study findings vary as to levels of empathy of students in other health care disciplines than physiotherapy. McKenna et al. found no differences in levels of empathy of undergraduate nursing students relating to study year.^15^ A study in Pakistan showed no difference in the levels of empathy between the first and fifth year medical students.^4^ Kimmelman et al. found that empathy of osteopathic medical students did not decrease significantly by study years.^16^ In our study also, no difference was found between the first and last years.

Wang et al. found small differences in the empathy scores of medical students in their different years of study,^9^ whereas other studies among medical students showed decreased levels of empathy.^17^,^18^ Empathy scores of Iranian medical students had a negative relationship with study years.^13^ Likewise, Sherman and Cramer found that first-year dentistry students had higher empathy levels than students in later years.^19^ The longitudinal study of Ward et al. also showed a decline in levels of empathy among nursing students.^20^ Conversely, other studies found higher empathy scores of students in their final year compared to those in their first year.^21^,^22^ Another study conducted at a medical college reported that while empathy scores did not alter during the first two years, they decreased during the 3rd year and remained low until graduation.^23^

The different studies cited above show that levels of empathy may either remain stable or increase or decrease. This variety may be a result of the different professions examined, different educational curricula, or cultural differences.

Wilson et al. showed that across the study years, empathy increased among pharmacy students, decreased among nurses, and remained the same among law students.^24^ Comparing levels of empathy of students in different health professions, paramedic trainees were found to have lower empathy scores than those in midwifery, occupational therapy, physiotherapy, medicine, or nutrition and dietetics. Williams et al. found that empathy scores of physiotherapy students are higher than those of other health professionals.^7^ Unfortunately, we did not compare our results with the other professions.

Our study is the first to target empathy in the discipline of physiotherapy. In this study, empathy scores did increase slightly after school entrance and during the 2nd and 3rd study years of training, but decreased significantly in the final year, which was primarily clinical practice. Perhaps levels of empathy rise year by year with classroom training, but diminish in the clinical phase of their education in their last year as the students learn to become more emotionally detached towards their patients in order to be coolheaded. Further relevant studies are needed on how and why empathy changes during the training of physiotherapy students, and also to help determine how empathy might be throughout the four years of physiotherapy programs. In any case, the curricula of physiotherapy departments of universities in Turkey, at least, should incorporate ways to increase empathy and communicational skills.

This study is important because it compares the results of two different kinds of universities, one governmental and the other a foundation school. Gabard et al. said that levels of empathy may differ between different institutions,^11^ but in our study, the difference between empathy scores of physiotherapy students from BVU and MU was borderline significant. As to study years, at both universities the difference of empathy scores was significant only at the 1st and 4th years. While levels of empathy of the students of the foundation university were higher than at the government university in the 1st year, those of the last year students at the foundation university decreased more than at the government university. Future studies may examine levels of empathy of the students who begin university and what in the curriculum adds to their levels of empathy of the same students.

We studied levels of empathy according to field of work, home region, and gender. Some studies showed that levels of empathy were higher among students who chose future interest specialization than among those who did not.^4^,^23^ On the other hand, Hasan et al. also found that desired specialty did not significantly associate with levels of empathy.^25^ In our study, the first two fields of choice were pediatric rehabilitation and neurologic rehabilitation. No relationship was seen between field of work and empathy scores. Students typically determine their specialty in the last year, because before that they are not sufficiently familiar with the various areas of specialization. The study of empathy is wide open for further research. Studies would be useful that show which physiotherapy specialties require more empathy.

Most of physiotherapy students of both universities in our study came from the Black Sea region. No differences was seen as to levels of empathy related to home-region, but further community-based studies might show differences of levels of empathy among those from various regions in Turkey.

Another area we studied was gender. Our study found no statistically significant difference in empathy scores between males and female physiotherapy students, but in the literature, female medical students are generally considered to be more empathetic than their male counterparts.^3^,^22^,^25^ Further research may determine if females are more perceptive to emotions and generally give significance to developing inter-personal relationships with patients, and if males take a more rational rather than emotional approach.

### Limitations of the study

This study was conducted at only two of the almost 60 physiotherapy departments in Turkey. Since the results may not necessarily be representative of physiotherapy students in general, a larger more wide-ranging study population is needed to validate our results. By studying physiotherapy departments of other universities in Turkey, the overall curriculum might be more appropriately improved to raise awareness of the importance of empathy, and give students specific opportunity to practice this valuable attribute. Also of interest is to measure how empathy varies each year throughout the four years of a typical physiotherapy program, following the same students in a prospective longitudinal rather than a cross-sectional study design. Finally, we need measurement methods which test practice-based empathy.
